# From assemblies to ancestry: Genomic advances illuminates legume evolution

**DOI:** 10.1590/1678-4685-GMB-2025-0245

**Published:** 2026-07-17

**Authors:** Thiago Nascimento, Andrea Pedrosa-Harand

**Affiliations:** 1 Universidade Federal de Pernambuco, Centro de Biociências, Departamento de Botânica, Laboratório de Citogenética e Evolução Vegetal , Recife, PE, Brazil.

**Keywords:** Ancestral karyotype, dysploidy, genome size, polyploidy, WGD - whole genome duplication, whole genome sequencing, repetitive sequences

## Abstract

Legumes are diverse, wide-spread and important both ecologically and economically. These features prompted the establishment of hundreds of genome assemblies as resources to investigate key biological questions. In this review, we introduce the reader to the Fabaceae (Leguminosae) family highlighting the importance and great diversity of this group of plants. The major insights gained from studying genomic variation in the legumes are discussed, with focus on whole genome duplications, ancestral chromosome numbers and key biological novelties such as the ability to fix nitrogen through symbiosis with bacteria.

## Introduction

**Figure 1 -:**
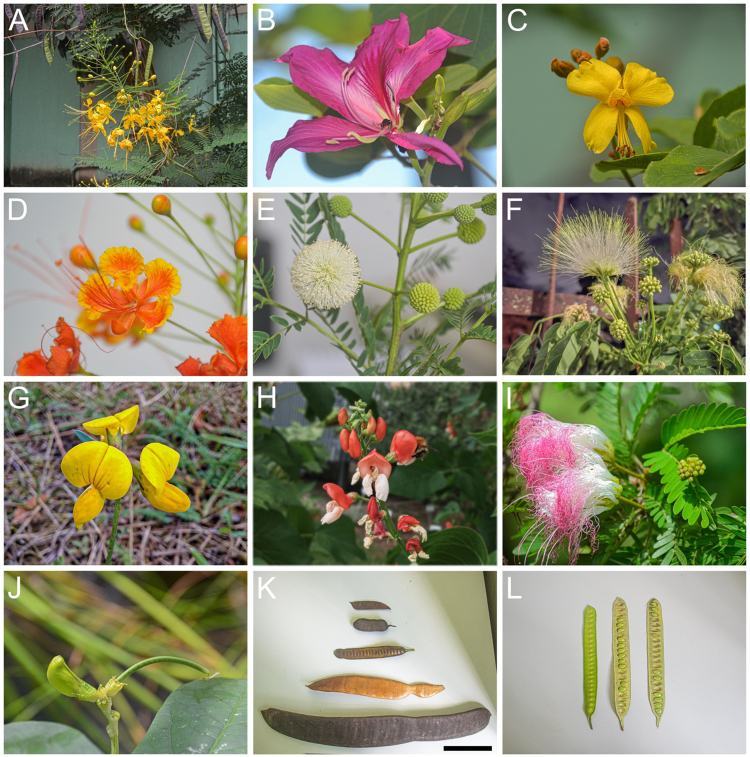
Morphological diversity in Leguminosae. (**a-d**) *Caesalpinia pulcherrima*, (**b**) *Bauhinia forficata*, (**c**) *Cenostigma nordestinum*, (**e**) *Leucaena leucocephala*, (**f**) *Albizia lebbeck*, (**g**) *Lotus corniculatus*, (**h**) *Phaseolus coccineus*, (**i**) *Calliandra surinamensis*, (**j**) *Phaseolus lunatus*. (**k**) Diversity of legume fruits (scale bar = 10 cm). (**l**) Typical seed organization within legume fruits.

The Leguminosae family (Fabaceae) is globally distributed, comprising 19,500-22,000 species distributed in 770-807 genera (LPWG, 2017; POWO, 2025), with at least 100 new species being catalogued every year. Members of this family occupy nearly all biomes and exhibit remarkable morphological, ecological, and life-history diversity (Figure 1). In the last five decades, this diversity was characterized in collections such as the Advances in Legume Systematics series, with the first volume (Polhill and Raven, 1981) published after the international Legume Conference at the Royal Botanic Gardens (Kew, England, July 1978), and the renowned book Legumes of the World (Lewis, 2005). More recently, the Legume Data Portal (http://www.legumedata.org) was created to unify community-endorsed occurrence, taxonomy and phylogenetic data for the family.

Legumes provide proteins and other nutrients, benefiting human health (Lewis, 2005; LPWG, 2013, 2017). Furthermore, due to the remarkable ability of fixing atmospheric nitrogen, legumes have a key role in the development of agriculture and the regulation of ecosystems, since this process enriches soil with nitrogen, reduces the use of synthetic fertilizers and increases yields (Kakraliya et al., 2018; Mathesius, 2022). In addition, legumes can also improve carbon sequestration, enhance nutrient cycling, support crop rotation diversification and emit fewer greenhouse gases per unit area than fertilized non legumes (Stagnari et al., 2017; Mathesius, 2022), underpinning their central roles in agriculture, ecosystem nutrient cycles, and sustainable food systems (Gepts et al., 2005; LPWG, 2017).

Legume research began with classical taxonomy studies in the 18th-19th centuries by Augustin Pyrame de Candolle and George Bentham and later progressed through comprehensive morphological treatments and floras into the 20th century (Harms, 1907, 1913; Martin, 1914). From the late 20th century, the use of plastidial and nuclear molecular markers (*rbcL*, *matK* and ITS, the Internal Transcribed Spacer of the 35S rDNA *locus*) reshaped legume systematics, prompting revisions at the genus and higher taxonomic levels (LPWG, 2013; Hughes et al., 2022; Bruneau et al., 2024). During the 1990s-2000s, molecular genetics expanded alongside the rise models for the genomic area, such as *Lotus japonicus* L. and *Medicago truncatula* L. (Handberg and Stougaard, 1992; Barker et al., 1990). Transcriptomes and early genome sequences enabled comparative analyses and translational applications in crop improvement (Gepts et al., 2005; LPWG, 2013). Collaborative efforts, particularly by the Legume Phylogeny Working Group (LPWG), enabled dense plastid (*matK*) and multilocus sampling to generate a nearly complete family-level phylogeny (LPWG, 2013, 2017). More recently, phylogenomics applying target-capture DNA sequencing and transcriptomic data have expanded sampling including herbarium materials, making possible a better understanding of the evolution of the family (Koenen et al., 2020). Also, the advances in sequencing technologies in the last decades is providing reference genome assemblies that, associated with integrative bioinformatic approaches, have the potential to solve multiple questions (Satam et al., 2023).

The recent large-scale phylogenetic studies have shown that the traditional three-subfamily classification (Caesalpinioideae, Mimosoideae and Papilionoideae) does not reflect the evolutionary history of legumes. In particular, the traditional Caesalpinioideae subfamily was found to be paraphyletic, leading to a reclassification that recognizes six monophyletic subfamilies: Cercidoideae, Detarioideae, Duparquetioideae, Dialioideae, a recircumscribed Caesalpinioideae (including the Mimosoid clade), and Papilionoideae (LPWG, 2017). The Papilionoideae subfamily is the largest clade in Leguminosae and concentrates almost all economically important crops of the family, including soybean [*Glycine max* (L.) Merr.], common bean (*Phaseolus vulgaris* L.), pea (*Pisum sativum* L.), chickpea (*Cicer arietinum* L.), lentil (*Lens culinaris* Medik.), and peanut (*Arachis hypogaea* L.).

With this robust phylogenetic framework as background, this review synthesizes current advances in legume genomics, with emphasis on its contribution to the understanding of legumes evolution. We discuss the genomic variation observed in the family, the efforts undertaken so far for making complete reference genome assemblies available, the synteny relationships between these available genomes and the efforts towards the reconstruction of an ancestral genome. We also discuss the contribution of these advances for central questions of legume plant biology, such as the ability to nodulate.

## Legumes diversification and genomic variation

One of the main evolutionary questions related to legumes is to understand the high rates of diversification of the group, considered the third largest family of flowering plants, only after Asteraceae and Orchidaceae. A large number of legume fossils have been found (Crepet and Herendeen, 1992; Herendeen and Dilcher, 1992; Wing et al., 2009), mostly dating from ~65.3 Mya during the Paleocene, right after the Cretaceous-Paleogene (K-Pg) mass extinction event caused by the impact of an asteroid on the coast of Mexico. These fossils enabled the reconstruction of a time-calibrated phylogenetic framework, revealing that legumes experienced rapid diversification and global radiation accompanied by multiple ancient Whole Genome Duplication (WGD) events, a process that leads to polyploidy. At least five such events, several occurring near the K-Pg boundary, occurred in the legume ancestor or, more likely, within its major subfamilies and likely played a key role in the survival and evolutionary success of the clade (Koenen et al., 2021; Zhao et al., 2021; Stai et al., 2026). In addition, a recent investigation has presented a high correlation between paleopolyploidization and the K-Pg event, which suggests an important role of polyploidy to the survival of organisms after a large environmental crisis (Chen et al., 2025). Also, WGD events can be associated with shifts of diversification, which could explain the high level of diversity among legumes (Landis et al., 2018). Although a single WGD event has been postulated on the ancestral of the whole family (Koenen *et al*. 2021; Zhao *et al.* 2021), recent evidence support distinct WGD events in each subfamily, with allopolyploidy (genome duplication after hybridization) likely occurring in Cercidoideae, Dialoideae, and Caesalpinioideae. The involvement of different progenitors obscure reconstruction of the legume phylogenetic backbone by introducing conflicting evolutionary signals (Stai *et al*., 2026).

Most legume species are distributed in the tropical regions of the globe, and the temperate species are hypothesized to have colonized this region only after the transition from wood to herbaceous growth form (Li et al., 2013). What is a consensus is that multiple diversification events occurred during the evolution of the group, with recent radiations as the ones observed in the genera *Lupinus* (Nevado et al., 2016) and *Inga* (Schley et al., 2025). 

Although the diversity of the Leguminosae family have been investigated during the last years, there are remaining questions about its species inventory, geographical distribution and evolutionary relationships. Fernandes et al. (2025) analysed the gaps in these fields and estimated that at least 11% (around 2505 species) of the diversity of legumes are yet not described, which is consistent with the prediction that 10-20% of the vascular plant species are unknown (Joppa et al., 2011). The understanding of spatial distribution of at least 20% of the accepted legume species is compromised by the lack of geographic data; and at least 50% of all legumes miss DNA sequencing data, even for well-known molecular markers such as ITS, *matK*, *psbA*, *rbcL* and *trnL*, hampering a complete phylogenetic analysis of legumes evolutionary history (Fernandes *et al*., 2024).

The high rates of legume diversity are also reflected in the genomic variation present in the family. The analysis of chromosome number and ploidy level by cytogenetic and genomic approaches has helped to shed light onto this variation. Legumes vary in chromosome numbers from 2*n* = 12 (as in *Sesbania bispinosa* (Jacq.) W.Wight) to 2*n* = 208 (as in *Vachellia hebeclada* (DC.) Kyal. & Boatwr.; Bairiganjan and Patnaik, 1989; Santos et al., 2025). Variations in chromosome numbers are associated to events of polyploidy and dysploidy, such as in *Senna.* In this genus, *n* = 14 is the most common chromosome number, but variations are frequently observed due to polyploidy (*n* = 21, 28, 56) or dysploidy (*n* = 11, 12, 13), the latter corresponding to changes in chromosome number caused by chromosomal structural rearrangements (Cordeiro and Felix, 2017). Polyploidy occurred several times during the evolution of legumes, since their origin to recent lineages as in soybean, and it is pointed as a key drive of its diversification (Doyle, 2012). 

**Figure 2 -:**
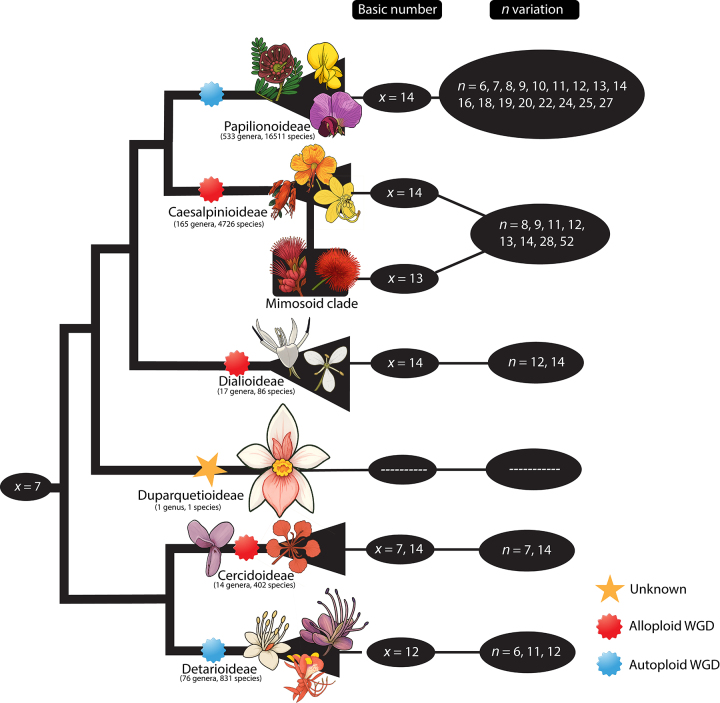
Phylogenetic relationships among Leguminosae subfamilies indicating basic chromosome number and chromosome number variation according to Stai et al. (2019). Flowers represent the morphological diversity of some representatives of each subfamily. Whole Genome Duplications (WGD) and topology are represented according to Stai *et al*. (2026). Red circles represent alloploid, while blue circles represent autoploid WGD, and the yellow star represents an unknown state.

For decades, the analysis of chromosome number was the only source of information for the study of karyotype evolution in legumes, with particular attention to the haploid chromosome number present in the gamete (*n*) and the basic chromosome number (*x*), which represents the ancestral chromosome number from which the species chromosome set (*n*) evolved. Goldblatt (1981) highlighted polyploidy and aneuploidy (possibly meaning dysploidy) cases across different groups of legumes and estimated the basic chromosome number *x* = 7 for the family, which became *x* = 14 in the Papilionoideae subfamily due to an ancient polyploid event. Subsequent chromosome reductions led to *x* = 6, 7, 8, 9, 10 and 11 for different groups within Papilionoideae. The basic number *x* = 7 proposed for Leguminosae was later endorsed by analyses of chromosome counts in the light of phylogenetic reconstruction, which calculated the modal gametic chromosomal count for Cercidoideae as *n* = 7, 14, Detarioideae as *n* = 12, Dialioideae as *n* = 14, and Caesalpinioideae as *n* = 12-14, with 13 for the Mimosoid clade (Figure 2). In Papilionoideae, the early-branching Papilionoideae lineages showed *n* = 13-14, while other groups varied from 7 to 11, as genistoids (9), dalbergioids (10), baphioids (11), mirbelioids (7-9), Robineae (10-11), Loteae (6-8), the IRLC clade (7, 8), indigoferoid (7, 8) and millettioid (11) (Stai et al., 2019; Ren et al., 2019). In this scenario, at least five WGD events influenced the chromosome number evolution in the family (Stai *et al*., 2019, 2026). These WGD events were also supported by a broader phylotranscriptomic and phylogenomic analysis, but an additional WGD event at the base of Leguminosae was also postulated, as well as more recent duplications and triplications in a total of 28 WGD/WGT events in the family (Zhao et al., 2021). This additional WGD shared by all legumes is controversial. It was also dated to the K-Pg boundary, as the subfamily-specific WGD events, and would imply, for instance, a basic number of *x* = 28 for Papilionoideae, what is not supported by other evidence.

A recent large-scale cytogenetic analysis of Caesalpinioideae species (Santos et al., 2025) highlighted the remarkable variation in chromosome number, heterochromatin organization, and genome size within the subfamily. Despite the wide range in chromosome numbers-from 2*n* = 14 to 2*n* = 208-the most common number across the clade are 2*n* = 26 and 28. Based on a phylogenetic framework, which suggested a diversification time of approximately 64.3 Mya for the subfamily, the authors reconstructed a basic chromosome number of *x* = 14. The results indicated that polyploidization, followed by both ascending and descending dysploidy, has been the primary driver of karyotype evolution in Caesalpinioideae.

Another way of accessing the genomic variation in plants is through the estimation of their genome size by techniques such as flow cytometry. Genome size estimations can capture the large existent variation among plants, which ranges from 0.0623 picograms (1C = 60 Mbp) in the carnivorous plant *Genlisea tuberosa* Rivadavia, Gonella & A. Fleischm (Fleischmann et al., 2014) to 164 pc (1C = 160.45 Gbp) in the Caledonian fork fern (*Tmesipteris oblanceolate* Copel.) (Fernández et al., 2024). For legumes, according to the Plant DNA C- values database provided by the Kew Botanic Garden (https://cvalues.science.kew.org/), the DNA amount vary from 1C = 0.28 pg (1C = 274 Mbp) in *Lotus unifoliolatus* to 1C = 27.40 pg (1C = 26.8 Gbp) in *Vicia faba*, a 97-fold variation. This variation in size can be explained by polyploidy and by the differential accumulation of the repetitive fraction, mostly by the expansion of specific satellite DNAs or different lineages of retroelements, as the Ty1/copia and Ty3/gypsy LTR-retrotransposons (Macas et al., 2015; Vicient and Casacuberta, 2017).

Whole Genome Duplications (WGDs), or polyploidy events, are widespread in plants and other eukaryotes. By generating duplicated genetic material-genes and regulatory components-they enhance evolutionary potential and enable novel traits and innovations. WGDs can also provide short-term advantages such as stress tolerance, heterozygosity, and shifts toward selfing or asexuality, which may aid survival during environmental change (Van de Peer *et al*., 2009; Clark and Donoghue, 2018). In the evolutionary history of legumes, the existence of WGDs events has also helped to shape its variation in genome size and karyotype evolution. Genomic data have proposed an ancient papilionoid WGD (59 Mya), retained as paralogous blocks in many legume genomes (e.g., soybean, *Medicago*, chickpea), and a more recent, lineage-specific *Glycine max* (soybean) WGD (13 Mya), that doubled soybean gene content and produced widespread retained duplicates and fractionation, the loss of duplicate genes after whole genome duplication (Schmutz et al., 2010; Young et al., 2011). Genome duplication events were also proposed for other Papilionoideae, such as in *Lupinus*, where a triplication event at 25 Mya affected many species in the Genisteae clade (Hane et al., 2016). Other Papilionoideae, such as *Cajanus cajan* (L.) Huth (pigeon pea), lack a recent WGD but still show synteny in WGD-derived regions (Varshney et al., 2012). These ancient WGD signatures are also present in other genomes (e.g., lentil, chickpea, cowpea) as residual paralogous blocks and elevated duplicated fractions (Varshney *et al*., 2013; Lonardi et al., 2019; Ramsay et al., 2021). Allotetraploid crops such as cultivated peanut (*Arachis hypogaea* L.) experienced hybrid polyploidy, producing A and B subgenomes with homeolog pairs showing biased retention, subgenome dominance and differential expression (Zhuang et al., 2019).

Although there are multiple evidences of WGD in Leguminosae, two factors limit the discussion about the time of these events and whether they are shared or not among lineages: the small and limited number of genome assemblies available for the other subfamilies, except Papilionoideae, and the lacking of discussion about genome evolution in most part of the papers published, with some being restricted to the description of the assembly or focused in functional properties. As an example, for the Cercidoideae subfamily, only four genomes are currently available at the NCBI platform, the genomes for *Cercis canadensis* and *C. chuniana* (Griesmann et al., 2018; Gong et al., 2025), *Bauhinia variegata* (Zhong et al., 2022) and the marama bean (*Tylosema esculentum*) (Li and Cullis, 2025). For *Cercis* species, the first genome assembly publication is focused on the evolution of nodulation, while the more recent one only describes the assembly for *C. chuniana*, with no discussion. In the genome description of *Bauhinia variegata*, a comparison is made with the *C. canadensis* genome, revealing the presence of signatures for a specific WGD in *Bauhinia*. For the marama bean genome study, it was compared in the synteny level only with *B*. *variegata*, displaying several breaks of synteny (mostly inversions and translocations), while *Ks* analysis suggested that the species probably underwent multiple rounds of WGD, which could explain the complexity of its genome. 

## Genomic resources and their contributions to deciphering legume genomic evolution

**Figure 3 -:**
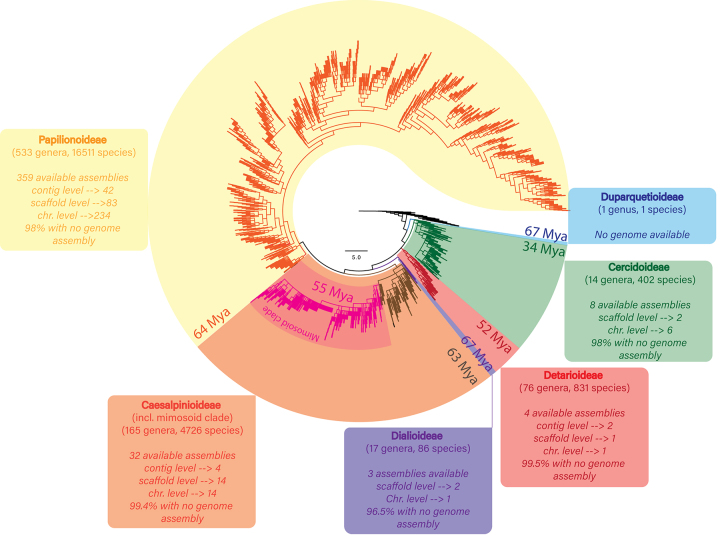
Available genome assemblies at different scales across Leguminosae subfamilies. Phylogenetic relationships are based on data from LPWG (2017), and divergence times follow Zhao et al. (2021).

According to the NCBI genome platform (www.ncbi.nlm.nih.gov/datasets/genome; accessed in October/2025), when considering only scaffold (S) and chromosome (C) scale genome assemblies at the time of writing, over 350 genome assemblies are publicly available for Leguminosae, which represents around 75 genera, covering all subfamilies except Duparquetioideae. These include Papilionoideae (54 genera, 320 assemblies, S=83, C=237), Caesalpinioideae (15 genera, 28 assemblies, S=14, C=14), Detarioideae (2 genera, 2 assemblies, S=1, C=1), Cercidoideae (3 genera, 4 assemblies, S=2, C=2), and Dialioideae (2 genera, 2 assemblies, S=1, C=1). When analysing the PubPlant database (https://www.plabipd.de/pubplant_main.html; Schwacke et al., 2025), which unifies only the genome assemblies associated with a formal publication, Papilionoideae still concentrates most studies (around 124 assemblies), followed by Caesalpinioideae with 23 assemblies, Cercidoideae (8), Dialioideae (2) and Detarioideae (1) (Figure 3). The disparity in genome availability reflects the larger number of species within Papilionoideae, but also the larger number of domesticated and economically important species within this subfamily. Although genome assemblies at scaffold level are important for the study of genes, here we focus the discussion mainly in assemblies at chromosome scale and their use in the analysis of the genomic evolution of the family and subfamilies.

The legume genomic era started with the draft genome assemblies for the model species *Lotus japonicus* and *Medicago truncatula*. *Lotus japonicus* is a widespread early-flowering plant (2*n* = 12), from the same genus as the polyploid *Lotus corniculatus*, a temperate forage, that has been used worldwide by scientists as a legume model, especially on the study of the mechanisms associated with nodulation (Ferreira and Pedrosa-Harand, 2014). The first genome assembly for the species was based in shotgun and clone-to-clone using BACs (Bacterial Artificial Chromosomes) and TACs (Transformation-competent Artificial Chromosomes) sequencing technologies, producing 44,464 contigs and approaching 315 Mbp of genome sequences, corresponding to ~67% of the total genome size previously estimated (475 Mb) and covering ~91% of the gene content (Sato et al., 2008). Despite limited in coverage, this first version of the genome has brought to light important features as the identification of specific genes, its incompleteness and the scarcity of other legume genomes, limiting deeper analysis on the evolution of the family. More recently, a high quality genome assembly using an association of Illumina, Pac-Bio and Hi-C (chromatin conformation) data has been published for *L. japonicus*, providing a more complete chromosome scale version with 544 Mb in size (357 Mb being repeats) and 27,911 annotated genes, making possible more accurate analysis and the comparison to other legume assemblies available as *Phaseolus vulgaris*, *Vigna radiata*, *Vigna angularis*, *Glycine max* and *Cicer arietinum* (Li et al., 2020). 

*Medicago truncatula* has been chosen as a model legume mostly due to its fast vegetative growth, small diploid genome size (different from its close related crop *M. sativa*, which is a tetraploid) and its low requirement for cultivation factors as temperature and light. The first draft genome for *Medicago truncatula* was released by Young et al. (2011), also based on conventional cloning approach using BACs followed by shotgun sequencing, capturing ~94% of *M. truncatula* genes. During the following years this version was improved, with new releases being published in 2014 (Tang et al., 2014) and 2018 (Pecrix et al., 2018). This last one is the most recent version, which was generated through Pac-Bio long read sequencing approach, accomplishing high levels of completeness and making possible to identify rearrangements in base-pair resolution among different genotypes.

Among other legume genomes assemblies available so far, the majority belongs to the Papilionoideae family, mainly focusing on economic important crops such as soybean (*Glycine max* L.), common bean (*Phaseolus vulgaris* L.), cowpea [*Vigna unguiculata* (L.) Walp.], chickpea (*Cicer arietinum* L.), lentil (*Lens* spp.) and peanuts (*Arachis hypogaea* L.). The soybean genome (~1.1 Gb in size and ~46,430 genes annotated) was one of the first reference genome for legumes and helped to evidence two WGD events, an ancient one in the Papilionoideae clade and a specific one in *Glycine* lineage, occurring ~56.5 and ~10 million years ago, respectively (Schmutz et al., 2010). Extensive paralog retention, and gene-fractionation patterns critical for trait mapping and polyploid evolution were reported (Schmutz *et al*., 2010). The draft genome assembly for chickpea (~738 Mb in size, ~28,269 genes annotated) provided a catalogue for resistance genes to many plant pests and pathogens and identified SNPs (Single Nucleotide Polymorphism) and SSRs (Simple Sequence Repeats) markers with potential to support genomic breeding in this orphan crop. When compared to other legume genomes available at that time, as *M. truncatula*, *L. japonicus*, pigeon pea [*Cajanus cajan* (L.) Huth] and soybean, chickpea presented an extensive conservation of synteny (around 90%) (Varshney et al., 2013). 

The common bean reference genome assembly (~474 Mb in size) enabled synteny analysis with the previous soybean genome. According to the measurement of accumulated mutations, both species diverged ~19.2 Mya, suggesting that *P. vulgaris* evolved faster than *G. max* (Schmutz et al., 2014). When comparing the synteny of both genomes, most of the ortholog genes between the species were syntenic, except for the pericentromeric regions, which presented breaks of microcolinearity. Due to the specific WGD in soybean, most of the genomic blocks in the common bean could be found twice in soybean genome, and the presence of more than 90% of *P. vulgaris* genes in *G. max* is probably a result of the shared ancient WGD. Additionally, the analysis of the common bean genome assembly revealed distinct selective sweeps for the two pools of diversification (Mesoamerican vs. Andean) associated with the two events of domestication and provided specific regions of the genome that can be used in the future studies as targets to crop improvement (Schmutz *et al*., 2014). In addition, a reference genome was assembled for cowpea (*Vigna unguiculata*), with the identification of ~29,773 protein-coding loci and candidate genes responsible for the process of domestication and the control of organ size (Lonardi et al., 2019). 

Chromosome-scale assemblies for cultivated and wild lentils (*Lens culinaris*, ~3.8 Gb, ~39,778 genes; *L. ervoides*, ~2.9 Gb, ~37,045 genes) revealed repeat expansion, structural rearrangements, and insights for introgression breeding (Ramsay et al., 2021). And the allotetraploid peanut (*Arachis hypogea*, 2*n* = 4*x* = 40) haplotype resolved genome assembly (~2.5 Gb, ~83,709 genes) characterized its A and B subgenomes, shedding light on homeolog dynamics, resistance-gene repertoires, and domestication-related gene-family changes (Bertioli et al., 2016, 2019; Zhuang et al., 2019). A reference genome assembly, although not at chromosome-scale, was generated for *Stylosanthes scabra* (2*n* = 4*x* = 40), a relative of peanut which is an orphan legume from the Caatinga biome in Brazil, a region characterized by its long dry season. The analysis of the genome identified 22,681 protein-coding genes that corresponded to 41% of the genome content and the authors discussed the role of specific genes in the adaptation of the plant to the environment, especially genes associated with resistance to pathogens and to drought (Ferreira-Neto et al., 2023). 

The first pea (*Pisum sativum;* 2*n*=14) reference genome (Kreplak et al., 2019) was a great advance in the genomic era for legumes, especially considering the challenge of sequencing and assembling genomes with a large size (1C = ~4.5 Gbp) and for its importance as the first model plant studied by Gregor Mendel. Approximately 44,756 genes were identified and annotated, however the largest fraction of the genome (~83%) has found to be composed of repetitive elements, in particular long terminal repeats (LTR) retrotransposons, estimated to be the main source of genome size expansion in the species due to the ability of these retrotransposons self-copying and move across the genome. The author also brought evidence that suggests a higher rate of genomic evolution in comparison with other legumes. More recently, an improved reference genome was proposed for pea (Yang et al., 2022), with larger contiguity specially for the repetitive regions but also bringing to light insights about the function of genes postulated more than one century ago by Mendel, mostly related to seed shape and stem length. Another legume with a giant genome is Faba bean [*Vicia faba* L. (2*n*=12)], with a genome size three times higher than pea (~13 Gbp). A reference genome for the species has also been recently assembled (Jayakodi et al., 2023), and the size variation could not be explained by any recent WGD event in *V. faba*. A deeper investigation of its repetitive fraction revealed that, similarly to pea, the faba bean genome passed through multiple expansions promoted by LTR-retrotransposons and tandem repeats (satellite DNAs), specially from the Ty3-gypsy-Ogre family, that alone comprised 44% of the total genome size and a diverse number of satellite tandem repeats (9.4%), mostly distributed at the pericentromeres. 

The other subfamilies of Leguminosae are less represented in genomic efforts. The Caesalpinioideae subfamily counts with only 28 genome assemblies available so far (scaffold or chromosome scale), representing only 9% (15) of the 165 genera described for the clade. Among those, we can highlight the genome assembly of *Senna tora*, which is widely used by its medicinal properties (Kang et al., 2020). Specific gene families related to the anthraquinone biosynthesis were identified, and it constitutes the first reference genome for the genus *Senna*, which is marked by polyploidy and dysploidy events.

Similar efforts have recently provided haplotype resolved chromosome-scale genome assemblies for seven species of economically important trees from Caesalpinioideae subfamily (including species from the Mimosoideae clade) as *Acacia confusa* (566 Mbp), *Delonix regia* (580 Mbp), *Mimosa bimucronata* (641 Mbp), *Albizia julibrissin* (705 Mbp), *Biancaea sappan* (872 Mbp), *Gleditsia sinensis* (921 Mbp) and *Leucaena leucocephala* (1.3 Gbp) and further comparison analysis that helped to bring insights about the polyploidization history of the group (Chen et al., 2024). The authors identified and discussed that after the WGD event shared by all Caesalpinioideae around 72 Mya, additional two recent events (16.2-19.5 and 7.1-9.5 Mya) happened in *L. leucocephala,* which was characterized as an octoploid (2*n* = 8*x* = 112) that passed through gene loss (~40%) and contractions of its genome size, mainly promoted by diploidization processes. Also, a larger scale phylogenomic analysis with seven more available genome assemblies suggests that the species belonging to the Mimosoideae clade have evolved faster than the remaining species from Caesalpinioideae. Since the basic number proposed for Mimosoideae clade and most species of Caesalpinioideae is *x* = 13 or 14, the authors analysed the macrosynteny among species. The results revealed that species with *n* = 13 diverged later in comparison to the species with *n* = 14 and the chromosome structure appeared to be more conserved among Mimosoid than in remaining Caesalpinioideae. More interestingly, the authors identified two chromosomes in species with *n* = 14 that fused to one chromosome in the karyotype with *n* = 13. Other genome assemblies are being generated for the subfamily, as the first genome assemblies for *Inga leyocalycina* and *Inga laurina*; Schley et al., 2024) and *Acacia* (*Acacia pycnantha*; McLay et al., 2022), this last one considered the largest plant genus spread in Australia. Although, according to the PubPlant platform, *Acacia* is the genus with more genome assemblies available for Caesalpinioideae (at least 5), there is still not a wide genomic comparison for the genus, which should be a target for future studies.

When we consider the other subfamilies, Detarioideae has only two genome assemblies available at scaffold and chromosome scale, corresponding to less than 1% of the species diversity already described for the clade. The available assemblies are for *Eurypetalum unijugum* and *Sindora glabra* (Yu et al., 2022)*,* however the first one is not associated to a formal published study and the second one is mentioned by the authors as belonging to the Caesalpinoideae subfamily, not bringing any discussion about Detarioideae. In Cercidoideae, only four genome assemblies are available so far at the NCBI databank (also less than 1% of the species described), in scaffold or chromosome scale. According to the PubPlant database, four further genomes are published, but at contig scale. Among those assemblies, the majority is for species belonging to *Cercis* (3) and *Bauhinia* (3). In *Cercis*, the species analysed so far were *C. canadensis*, *C. chinensis* and *C. chuniana* (Griesmann et al., 2018; Li et al., 2023; Gong et al., 2025), however two of these studies limited the discussion to the genomic evolution of the genus or the subfamily, focusing on the description of the assembly or in the genes related to nodulation. The synteny analysis among *C. chinensis*, soybean, and coffee did not reveal evidence of WGD in the *C. chinensis* genome. This result challenges the hypothesis of a WGD event in the common ancestor of the Fabaceae family, instead supporting the interpretation that WGD events occurred independently in each subfamily, or at least after the divergence of the Cercidoideae subfamily. These results were further corroborated by other analysis including *Cercis* and non-*Cercis* species, which support the single hybridization hypothesis that led to the origin of all Cercidoideae and argue against a legume wide WGD event, with WGD events restricted to each subfamily (Stai et al., 2026).

For the *Bauhinia* genus, the three species with genome assemblies available so far are *B. variegata*, *B. purpurea* and the hybrid between both species, the Hong Kong orchid tree *Bauhinia* × *blakeana* Dunn (Zhong et al., 2022; Mu et al., 2025), this last one being one of the most complete genomes assemblies (phased by haplotype and telomere-to-telomere) available for legumes. In the more recent study, the *Bauhinia* species are compared in the genomic context with other legumes (including *Cercis* and model species as soybean, *L. japonicus* and *M. trucatula*), however only in the context of retraction and expansion of the specific genes (especially the ones related to resistance and terpene synthesis). The other two genomes for Cercidoideae are the haplotype phased genome of *Phanera championii* (Lu et al., 2024) and the genome of marama bean (*Tylosema esculentum;* Li and Cullis, 2025). The comparative genomics of *P. championii*, mostly with species from Papilionoideae clade, revealed that the species experienced two rounds of WGDs, one ancient event that corresponds to the whole genome triplication (WGT) of the core eudicot ancestor and a more recent WGD in Cercidioideae after the divergency of *Cercis*, with ~59.6% of its genes originating after these events. The marama bean genome analysis indicated a phylogenetic proximity to *Cercis* and *Bauhinia*, plus an ancient duplication event. 

In Dialioideae, the two genome assemblies available represent two of the 17 genera described for the subfamily, corresponding to only ~2.3% of the 86 described species. Among the assemblies, only one is at chromosome scale, from *Zenia insignis* (Chen et al., 2025), but it focuses on the description of the assembly. For *Dycorinia guianensis*, the scaffolds are phased with one assembly to each of its haplotypes (Schmitt et al., 2024) and the focus of the study is the transmission of low-frequency mutation. The Duparquetioideae subfamily is a special case, since it is composed by only one species (*Duparquetia orchidacea*) and its phylogenetic placement relative to Cercidoideae and Detarioideae is still an opened question as demonstrated by the minor differences in the relative placements of these lineages in different studies (LPWG, 2017; Stai et al., 2019, 2026; Aygoren Uluer et al., 2020; Zuntini et al., 2024). The genome organization of *Duparquetia* could give important insights into the evolution and diversification of the family. However, no genome assembly was generated so far for this monotypic subfamily and even general genetic information as genome size (1C value) or chromosome number are inexistent for the species 

With the advance of sequencing technologies in the recent years, allied to the decrease of their prices, it is expected an increase of the number of available genome assemblies in the following years. Long read sequencing technologies as PacBio HiFi associated or not with Hi-C data and Oxford Nanopore (ONT) have revolutionized genomics through the facilitation to generate high quality assemblies (Athanasopoulou et al., 2021; Mardis and Wilson, 2025), making available telomere to telomere (T2T) and haplotype phased reference genomes, as the one provided for the Hong Kong orchid tree *Bauhinia × blakeana* (Cercidoideae; Mu et al., 2025), and for model or economically important species from Papilionoideae subfamily as *M. truncatula* (Shen et al., 2025), soybean (accession ZH13; Zhang et al., 2023), common bean (*P. vulgaris* YP4; Wang et al., 2025 b ) and cultivated peanut (Wang *et al*., 2025a).

## Synteny and ancestral genomes

Papilionoideae, by far the largest subfamily, has been the most investigated so far, with most of the genome assemblies distributed among the tribes Phaseoleae, Dalbergieae, Robinieae and in species belonging to the clade with a loss of the chloroplast inverted repeat region (IRLC- Inverted Repeat-Lacking Clade, included in Papilionoideae). The reconstruction of ancestral genomes for the tribes, subfamily and family is still a big challenge, especially because plant genomes are prone to events as whole genome duplications (WGD) followed by diploidization involving structural rearrangements such as inversions, duplications, translocation, fusions and fissions.

Ren et al. (2019) proposed an ancestral genome for legumes based on syntenic blocks from selected legumes with genome assemblies available at that time (*Arachis duranensis*, *A. ipaensis*, *C. cajan*, *G. max*, *P. vulgaris*, *Vigna radiata*, *L. japonicus*, *M. truncatula* and *C. arietinum*). The authors discussed that the most recent common ancestor of the Papilionoideae probably had a haploid karyotype with nine chromosomes (*x* = 9). In this scenario, a putative single fusion reduced the chromosome number from *n* = 9 to 8 in *M. truncatula* and *C. arietinum*, diverse structural rearrangements reduced this number to *n* = 6 in *L. japonicus*, and fissions increased chromosome number to *n* = 10 and then to *n* = 11 in *Phaseolus* and *Vigna* species. Due to the particular WGD event that occurred around 10 Mya in *Glycine*, this number was doubled to *n* = 20. Considering the base chromosome number for the Leguminosae family as *x* = 7 (Goldblatt, 1981; Doyle, 2012), the haploid chromosome number *n* =14 proposed for the early diverged papilionoid is a result of the WGD event, with *n* = 9 being an intermediate stage during the diversification of the group. 

To investigate the genome evolution of specific clades or species, different ancestral genome predictions have been made in recent years. The analysis of the white lupin (*Lupinus albus* L.) genome in comparison to others 11 legume genomes predicted an ancestral legume karyotype composed of 16 conserved regions instead of only 14, although two putative chromosomes were significantly reduced in size (Hufnagel et al., 2020). The authors hypothesised that during the evolution of the white lupin genome, 15 chromosome fissions and 21 fusions resulted in the lupin ancestor with *n* = 9. The last experienced a whole genome triplication to result in the intermediate ancestral karyotype with *n* = 27. The establishment of the reference genome for pea (Kreplak et al., 2019) provided hypotheses for ancestral genomes for the family and for the Galegoid (pea, lentils, chickpea and faba bean) and Milletoid (common bean, cowpea and mung bean) clades through synteny analysis. These two clade-specific ancestral genomes, with *n* = 8 and *n* = 16, respectively, were proposed based on an ancestral legume karyotype with 25 synteny blocks arranged in 19 proto-chromosomes, higher than the putative *x* = 14 after WGD. The pea genome revealed specific breaks of synteny, usually associated to 5S rDNA sites, suggesting a role of this ribosomal DNA in these translocation events. 

The analysis of the peanut reference genome also supported the prediction of an ancestral legume karyotype, based on the genes of common bean corresponding to 16 pseudo-molecules. The comparison with this ancestral genome was used to unveil the evolutionary pathway of peanut karyotype. Six peanuts chromosomes originated from six fusions of twelve ancestral chromosomes, while the remaining four chromosomes were generated by two translocations among ancestral chromosomes. A and B subgenomes were later differentiated by translocation involving B7 and B8 chromosomes (Zhuang et al., 2019). 

In order to investigate the chromosome evolution and propose an ancestral genome for the tribe Phaseoleae, Montenegro et al. (2022) used a genomic block approach and hypothesized an Ancestral Phaseoleae Karyotype (APK) with *n* = 11 as a result from five end-to-end fusions of the 16 putative ancestral chromosomes proposed by Hufnagel et al. (2020), involving the two smallest blocks mentioned above, and in accordance with the previous basic chromosome number described to species belonging to this tribe (Ren et al. 2019). This analysis identified recurrent centromere repositioning events, particularly in common bean (*P. vulgaris* L.). More efforts are necessary to elucidate the ancestral karyotype of Leguminosae, since most chromosome scale assemblies available so far are provided only to the Papilionoideae subfamily and not even its putative 14 ancestral chromosomes are elucidated.

## Legumes biology: Nodulation

Legumes are well-known for their ability of forming a symbiotic complex with diazotrophic (capable of nitrogen fixing) bacteria, that are generally referred to as rhizobia, or with bacteria from the genus *Frankia*. These bacteria concentrate their activities inside of specialized structures located in the roots called nodules. Fixation of atmospheric nitrogen (N_2_) occurs by the action of nitrogenase enzyme that catalyses nitrogen molecules into ammonia (NH_3_), which can be processed by the plant during photosynthesis (Sprent, 2009). This important trait was identified during the 19^th^ century, firstly with the description of rhizobia and then *Frankia* activities (Hellriegel and Wilfarth, 1888; Hiltner, 1895), opening the discussion about the scattered and restricted distribution of nodulation among flowering plants. The mechanisms of infection used by N-fixing bacteria are diverse and the structures formed in the roots and nodules also vary (Xu and Wang, 2023).

Nodulation seems to have evolved independently in different clades during evolution. The phylogenetic distribution of nodulating plants indicates that both nodulating and non-nodulating lineages are nested within a single monophyletic group-the nitrogen-fixing clade-comprising representatives of the orders Fabales, Fagales, Cucurbitales and Rosales. Although each of these orders is itself monophyletic, nodulating taxa are phylogenetically dispersed within the clade rather than forming a single lineage, consistent with phylogenomic evidence for multiple independent origins of nodulation (Soltis et al., 1995). Among the six legume subfamilies, the species within Cercidoideae, Detarioideae, Dialioideae and Duparquetioideae are considered as non-nodulating, restricting the nodulation to a single Papilionoideae + Caesalpinioideae clade (Ardley and Sprent, 2021). But the non-legume lineage *Parasponia* from the Cannabaceae (Rosales) family, for instance, is also capable of hosting rhizobia bacteria and processing atmospheric nitrogen (Behm et al., 2014). 

The evolutionary origins of nodulation are still uncertain and in constant debate. To explain the interspersed distribution of both lineages, the most accepted hypothesis is that this character evolved through parallel evolution followed by successive losses by selection pressure (Griesmann et al., 2018; van Velzen *et al*., 2018, 2019; Zhao et al., 2021). However this hypothesis is still in debate, especially considering two aspects: parallel evolution itself cannot explain why all nodulating species are situated in the nitrogen-fixing clade without considering a common ancestor ~100 My that facilitated the process (Soltis et al.,1995; Werner et al., 2014), and also the conflict between several parallel origins and data about structure and development of nodulation (Swensen, 1996; van Velzen *et al*., 2019). Although recent phylogenomic analyses support up to 16 independent origins of nodulation (Kates et al., 2024), proponents of the single gain/massive parallel loss model argue that it remains plausible, proposing that the patchy absence of nodulation across lineages may reflect reversible phenotypic suppression driven by unfavourable carbon-nitrogen trade-offs rather than true evolutionary loss. In this scenario, prolonged cessation of nodulation would lead to stochastic genetic decay of unused symbiotic components-analogous to the degeneration of vision in cave-dwelling organisms-thereby creating patterns that may be misinterpreted as primary absence in phylogenetic reconstructions (Doyle et al., 2025).

The use of third generation sequencing technologies allied to transcriptomic data has increased the understanding of the mechanisms behind the evolution and function of nodulation. For example, the genome assembly for *Astragalus sinicus* and data about the expression of its genes revealed expansion of specific genes related to resistance and its primarily accumulation on the roots, enhancing roots protection during symbiosis (Chang et al., 2022). The improved version of the genome assembly of the model *Medicago truncatula* by PacBio sequencing and tissue-specific (roots and nodules) RNA sequencing promoted a better understanding of the genetic regulation of nodulation, characterizing the involvement of long non-coding RNAs (lncRNAs) in the symbiosis, plus the organization of the nodule expressed genes in genomic clusters (named by the author as symbiotic islands), which can be regulated by DNA methylation (Pecrix et al., 2018).

More recently, using extensive phylogenomic (88 species) and phylotranscriptomic (151 RNA-seq libraries) approaches, Zhang et al. (2024) provided an overview of the genetics of nitrogen-fixing root-nodule symbiosis. The authors found no evidence that individual genes evolved through convergence. Approximately 70% of the symbiosis-related genes identified were conserved across the species analysed, with particular attention given to the NIN gene, which has a unique role in nodulation. NIN is considered the best candidate gene specifically associated with nodulation and warrants further investigation. The study also raised the possibility that intermediate species may exist between nodulating and non-nodulating plants-species that possess only part of the genetic machinery required for nodulation. However, confirming this will require either identifying such species in nature or engineering them experimentally. Although these findings provide new insights into the evolution of nodulation, the question of its origin remains unresolved. This is particularly challenging given that nodulation fulfils similar functions in species that diverged more than 100 million years ago, despite involving different bacterial partners and structures. To test competing hypotheses-whether nodulation originated once from an ancestral symbiosis or evolved multiple times-broader large scale phylogenomic studies will be necessary.

## Perspectives and challenges 

Genomic studies in legumes offer unprecedented opportunities to address fundamental questions about plant evolution, crop domestication, and symbiotic interactions, but they also present significant challenges. The availability of hundreds of reference genomes, especially in Papilionoideae, has facilitated comparative genomics, shedding light on events such as whole-genome duplications, genome fractionation, and the evolution of nodulation. However, the genomic landscape of legumes remains uneven: while major crops and model plants as soybean, common bean and *M. truncatula* have high-quality chromosome-scale assemblies, most subfamilies-such as Cercidoideae, Detarioideae, Dialioideae, and the monospecific Duparquetioideae-are still underrepresented or lack genomic resources altogether. This imbalance limits broader phylogenomic insights and hampers the reconstruction of ancestral genomes that reflect the evolution of the family. 

Another challenge is the genome size variation, ranging from compact genomes to massive repeat-rich genomes such as pea and faba bean, which complicates sequencing and assembly efforts. Furthermore, despite advances in long-read sequencing and the availability of telomere-to-telomere assemblies, many published genomes still lack in-depth analyses and discussions related to repeat organization, structural variation, polyploidization history, and genome evolution, being restricted to the description of the assembly or catalogues of genes. Moving forward, perspectives in legume genomics involve expanding genomic coverage across neglected subfamilies, integrating comparative approaches to refine models of genome evolution, exploring the non-genic landscape, and leveraging pangenomics and functional genomics to bridge evolutionary biology with applied breeding. These efforts will be crucial not only to solve long standing evolutionary questions, such as the origin and loss of nodulation, but also to harness legume diversity for sustainable agriculture.

## Data Availability

This manuscript is a review article and does not include any new data generated by the authors. All data discussed are available in the published databases and literature cited throughout the text.
